# Gene Delivery to Nonhuman Primate Preimplantation Embryos Using Recombinant Adeno‐Associated Virus

**DOI:** 10.1002/advs.201900440

**Published:** 2019-09-04

**Authors:** Dan Wang, Yuyu Niu, Lingzhi Ren, Yu Kang, Phillip W. L. Tai, Chenyang Si, Craig A. Mendonca, Hong Ma, Guangping Gao, Weizhi Ji

**Affiliations:** ^1^ Horae Gene Therapy Center University of Massachusetts Medical School Worcester MA 01605 USA; ^2^ Department of Microbiology and Physiological Systems University of Massachusetts Medical School Worcester MA 01605 USA; ^3^ Yunnan Key Laboratory of Primate Biomedicine Research Institute of Primate Translational Medicine Kunming University of Science and Technology Kunming 650500 Yunnan China; ^4^ Li Weibo Institute for Rare Diseases Research University of Massachusetts Medical School Worcester MA 01605 USA

**Keywords:** adeno‐associated virus (AAV), animal modeling, CRISPR, genome editing

## Abstract

Delivery of genome editing tools to mammalian zygotes has revolutionized animal modeling. However, the mechanical delivery method to introduce genes and proteins to zygotes remains a challenge for some animal species that are important in biomedical research. Here, an approach to achieve gene delivery and genome editing in nonhuman primate embryos is presented by infecting zygotes with recombinant adeno‐associated viruses (rAAVs). Together with previous reports from the authors of this paper and others, this approach is potentially applicable to a broad range of mammals. In addition to genome editing and animal modeling, this rAAV‐based method can facilitate gene function studies in early‐stage embryos.

## Graphical abstract

**Delivery of genome editing tools to mammalian zygotes** has revolutionized animal modeling. However, the mechanical delivery method to introduce genes and proteins to zygotes remains a challenge for some animal species that are important in biomedical research. A viral vector infection approach to achieve gene delivery and genome editing in nonhuman primate embryos is presented in this study.

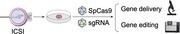

Delivering CRISPR reagents to mammalian zygotes for gene editing has greatly expedited the generation of genetically modified animals such as cynomolgus monkeys.^1^ However, the delivery of nucleic acids or proteins to preimplantation embryos mostly relies on laborious and time‐consuming procedures including microinjection as well as specialized equipment and techniques that collectively incur substantial expenses and turnaround. Furthermore, such mechanical procedures face species‐specific difficulties regarding practicality. For example, the bovine and porcine zygotes appear opaque due to lipid droplets, making the pronucleus not readily discernable for microinjection. Previously, we and others developed a simple and efficient method for gene delivery to mouse preimplantation embryos of various developmental stages using recombinant adeno‐associated virus (rAAV).^2^ This technique allowed us to conveniently deliver the Cre recombinase or CRISPR components to mouse zygotes by incubating with rAAV expressing the related genes, which enabled highly efficient genome manipulation and generation of genetically modified mice.^2^ We reasoned that rAAV may achieve gene transfer to zygotes of a broad range of mammalian species widely used in biomedical research. In particular, when this technique is coupled with CRISPR delivery for genome editing, it represents an appealing alternative technique for animal modeling that overcomes several difficulties associated with microinjection, and can be potentially applicable to various species commonly used as preclinical animal models.

In this study, we aim to demonstrate the feasibility of using rAAV for gene delivery to nonhuman primate (NHP) zygotes and genome editing. We chose cynomolgus monkey because it is a preferred NHP model in neuroscience research due to its similarities to humans in brain anatomy and function. We obtained zygotes by intracytoplasmic sperm injection (ICSI), and first screened rAAV of various serotypes expressing the enhanced green fluorescent protein (EGFP) for high transduction capability (Figure S1a, Supporting Information). We adopted the established rAAV treatment protocol for mouse embryos, and selected the AAV serotypes that perform well in mouse embryos, including AAV6, AAV6.2, AAV7, AAVrh.39, and AAVrh.43.^2^ NHP zygotes were incubated with 6 × 10^9^ genome copies (GC) of each rAAV for 5 h, rinsed, cultured for seven days ex vivo, and examined for EGFP fluorescence. Consistent with our previous mouse studies, all serotype vectors transduced NHP embryos, and rAAV6 performed slightly better than the others (Figure S1b,c, Supporting Information). However, in contrast to mouse embryos that can tolerate the same rAAV treatment regimen,^2^ NHP embryo development was severely compromised, resulting in most embryos stalling before the 8‐cell stage and no embryos progressing to blastocyst (Figure S1c, Supporting Information). We next used a tenfold lower dose of rAAV6 that was also highly effective in transducing mouse embryos,^2^ and tested various incubation durations ranging from 1 to 4 h (Figure S2a, Supporting Information). Approximately 60% of embryos developed into blastocyst, comparable to our typical yield of blastocysts when embryos were cultured under the same condition without rAAV. The 4 h incubation resulted in the most robust EGFP expression (Figure S2b,c, Supporting Information), although the EGFP signal was markedly weaker than the 6 × 10^9^ GC infection (Figure S1b, Supporting Information). Together, this initial set of experiments demonstrated the capability of rAAV to transduce NHP preimplantation embryos, and established an effective infection protocol.

To test whether rAAV infection of NHP zygotes can deliver CRISPR to realize genome editing, we chose to target the *ASPA* gene, mutations of which cause a fatal pediatric leukodystrophy called Canavan disease.^3^ We designed three sgRNAs targeting a coding region in cynomolgus monkey *ASPA* exon 2 that is downstream of the start codon (sgASPA) (**Figure** **1**a). The sgASPA targeting sequences were purposefully chosen to be conserved between cynomolgus monkey and African green monkey, so that the sgRNA targeting efficiency can be conveniently evaluated in the COS‐7 cell line that is derived from the latter species. Cotransfection of a pair of plasmids expressing SpCas9 and a single sgASPA (Figure 1b) in COS‐7 cells resulted in insertion/deletion (indel) mutations in the *ASPA* gene, and sgASPA2 yielded the highest frequency of indel mutations as quantified by TOPO cloning of the targeted amplicons and Sanger sequencing of individual clones (Figure S3, Supporting Information). Therefore, the SpCas9 and sgASPA2 constructs were separately packaged into AAV6 for coinfecting zygotes at 1:1 ratio (Figure 1c).

**Figure 1:**
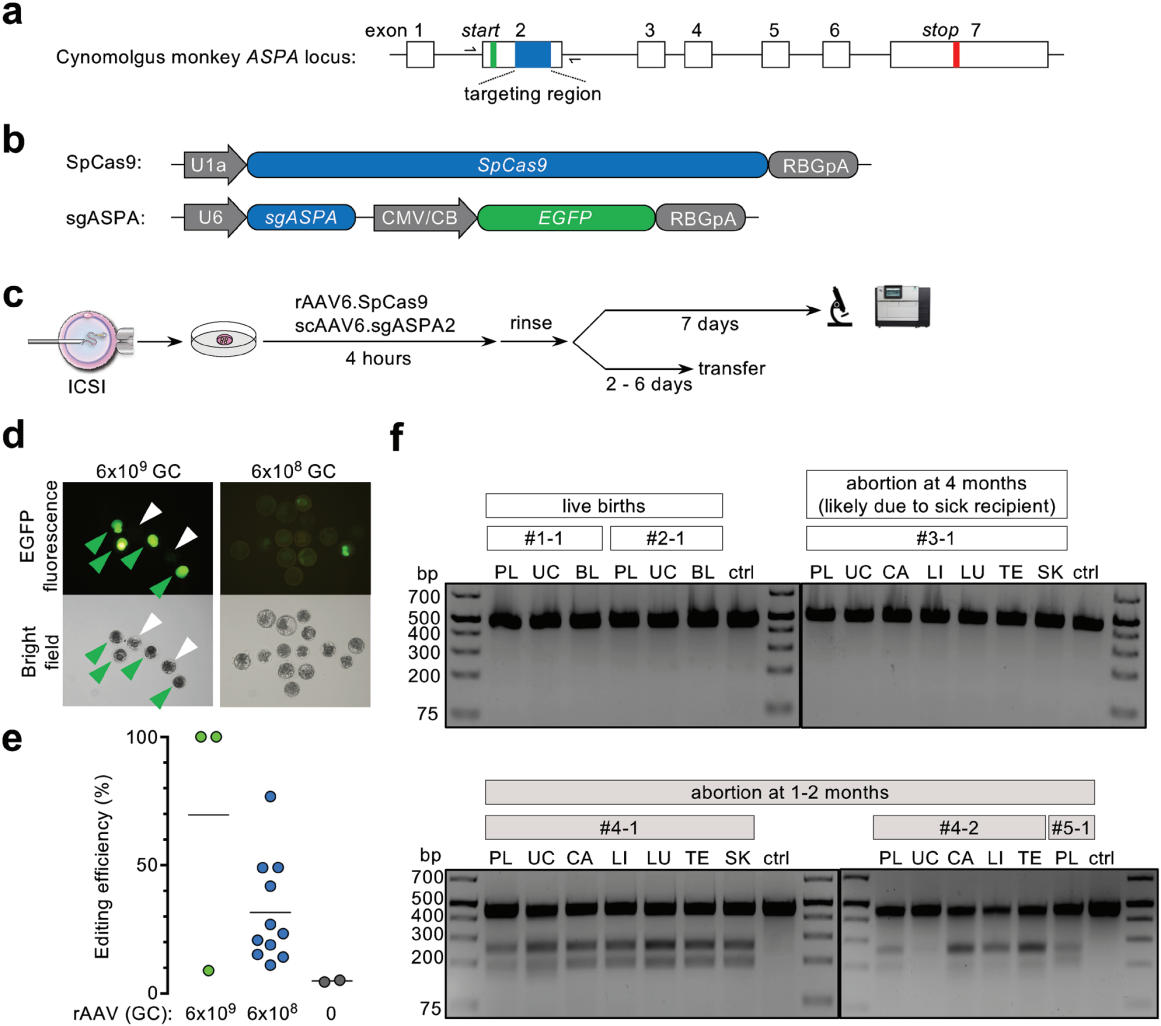
AAV vectors transduced preimplantation NHP embryos and achieved genome editing. a) The cynomolgus monkey *ASPA* gene locus. The sequences encoding the start and stop codons are indicated by the green and red bars, respectively. Three sgRNAs are designed to target a region downstream of the start codon within exon 2 (blue). Arrows denote PCR primer binding sites to generate amplicons for analyzing editing efficiency. Not drawn to scale. b) Schematics showing the constructs expressing SpCas9 and sgRNA, respectively. c) Workflow including intracytoplasmic sperm injection (ICSI), embryo infection, ex vivo examination, and embryo transfer. d) Microscopic images of NHP embryos infected with AAV6 vectors at two doses. Green arrowheads indicate transduced embryos, and white arrowheads indicate nontransduced embryos. e) *ASPA* gene editing efficiency of individual embryos as determined by SMRT sequencing. Horizontal bars indicate the average values. f) T7EI assay of DNA from various tissues of six subjects derived from rAAV‐infected embryos. PL: placenta (fetus side). UC: umbilical cord. BL: blood. CA: cardiac muscle. LI: liver. LU: lung. TE: testis. SK: skin. DNA from a naïve animal was used as control (ctrl).

Consistent with our initial experiments (Figures S1 and S2, Supporting Information), the high dose of 6 × 10^9^ GC compromised embryo development, whereas 6 × 10^8^ GC was tolerated (Figure S4, Supporting Information), and transduction was largely dose‐dependent as gauged by the EGFP fluorescence expressed from scAAV6.sgASPA (Figure 1d). A subset of embryos were individually harvested and analyzed for *ASPA* gene editing by single‐molecule real‐time (SMRT) sequencing. Two out of three embryos infected by the high‐dose rAAVs were found to be completely edited, while the embryos infected by the low‐dose rAAVs exhibited more variable editing efficiency ranging from 11% to 76% with an average of 31% (Figure 1e). Therefore, rAAV6 vectors can deliver CRISPR to NHP preimplantation embryos to afford targeted genome editing.

We further attempted to create cynomolgus monkeys with *ASPA* mutations, aiming to generate a NHP model of Canavan disease to further study the disease pathomechanisms and evaluate gene therapy.^4^, ^5^, ^6^ To this end, we infected another batch of zygotes with rAAV6.SpCas9 and scAAV6.sgASPA2 at 6 × 10^8^ GC total, and transferred the normally cleaved embryos to surrogate females. Three weeks later, ultrasonography revealed that five pregnancies were established carrying a total of nine fetuses. However, most fetuses underwent spontaneous abortion prior to full term. We obtained two live births and recovered four aborted fetuses at various developmental stages (Table S1, Supporting Information). Tissues from these individuals were collected and subjected to DNA analysis. *ASPA* gene editing was not detected in the live births (#1–1 and #2–1) and one fetus aborted at 4 months likely due to the sick recipient (#3–1), whereas three fetuses aborted at 1–2 months (#4–1, #4–2, and #5–1) were positive (Figure 1f). Despite the small sample size, the data indicated that *ASPA* mutations may be detrimental to cynomolgus monkey embryo development. However, we cannot rule out other possibilities leading to the early abortions including, but not limited to, rAAV infection and rAAV genome integration. Indeed, we previously detected rAAV genome integration in 1 out of 45 subjects in our mouse study.^2^ To characterize potential rAAV genome integration, we devised three PCR assays targeting different regions of the rAAV.U1a‐SpCas9 transgene cassette with sensitivities up to 1 in 500 haploid genomes (Figure S5, Supporting Information), and two PCR assays detecting potential integration of the AAV inverted terminal repeats (ITRs) at the on‐target *ASPA* locus with sensitivities up to 1 in 30 haploid genomes (Figure S6, Supporting Information). However, we did not detect rAAV genome integration in any tissue derived from the three gene editing–positive fetuses. In addition, the variation of gene editing efficiency among individuals suggests that not all zygotes were transduced equally despite being treated in the same droplet of culture medium, corroborating the transduction results measured by EGFP fluorescence (Figure 1d).

We next pursued to achieve a more robust gene editing efficiency using two sgRNAs. We generated another construct expressing both sgASPA2 and sgASPA1 (dual‐sgASPA) (**Figure** **2**a), and confirmed in COS‐7 cells that it could induce both local indels at the sgRNA targeting sites and large deletions in between (Figure S7, Supporting Information). A batch of seven zygotes were coinfected with rAAV6.SpCas9 and scAAV6.dual‐sgASPA at 6 × 10^8^ GC total, and individually analyzed for dual‐sgRNA‐induced large deletion by PCR (Figure 2b) and *ASPA* gene editing by SMRT sequencing (Figure 2c). Both assays revealed that 5 out of 7 embryos carried large deletions. Overall, the average gene editing efficiency was 77% with 5 out of 7 embryos exhibiting complete gene editing (Figure 2d), which compares favorably with using a single sgRNA at the same dose (Figure 1e). Furthermore, we examined the potential off‐targeting (OT) cleavage in these embryos. A total of 43 candidate OT sites of sgASPA1 (*n* = 35) and sgASPA2 (*n* = 8) were predicted using the Cas‐OFFinder bioinformatics tool (Tables S2 and S3, Supporting Information), mostly harboring three mismatches to the corresponding sgRNAs. Previous work suggested that mismatches distal to the protospacer adjacent motif (PAM) and fewer mismatches more likely result in OT cleavage.^7^ Therefore, we tested six most susceptible OT sites, i.e., those containing either three mismatches within nucleotides 13–20 upstream of the PAM (OT1, 2, 3, 5, 6) or two mismatches (OT4) (Figure S8a, Supporting Information). T7EI nuclease assay appeared “positive” for OT2 and OT5 (Figure S8b, Supporting Information). However, the DNA from a naïve embryo showed the same T7EI digestion patterns, suggesting irrelevance to Cas9‐induced indel mutations. Indeed, TOPO cloning and Sanger sequencing of the control amplicons of both OT2 and OT5 revealed dimorphic alleles that resulted in the T7EI cleavage patterns (Figure S8c, Supporting Information). Therefore, no off‐targeting cleavage was detected at the most susceptible candidate OT sites tested. This result was further corroborated by deep sequencing of the candidate off‐target PCR amplicons (Figure S8d,e, Supporting Information). In future animal modeling applications, a more comprehensive and unbiased off‐target analysis can be performed to screen for the most appropriate F0 animals as founders.

**Figure 2:**
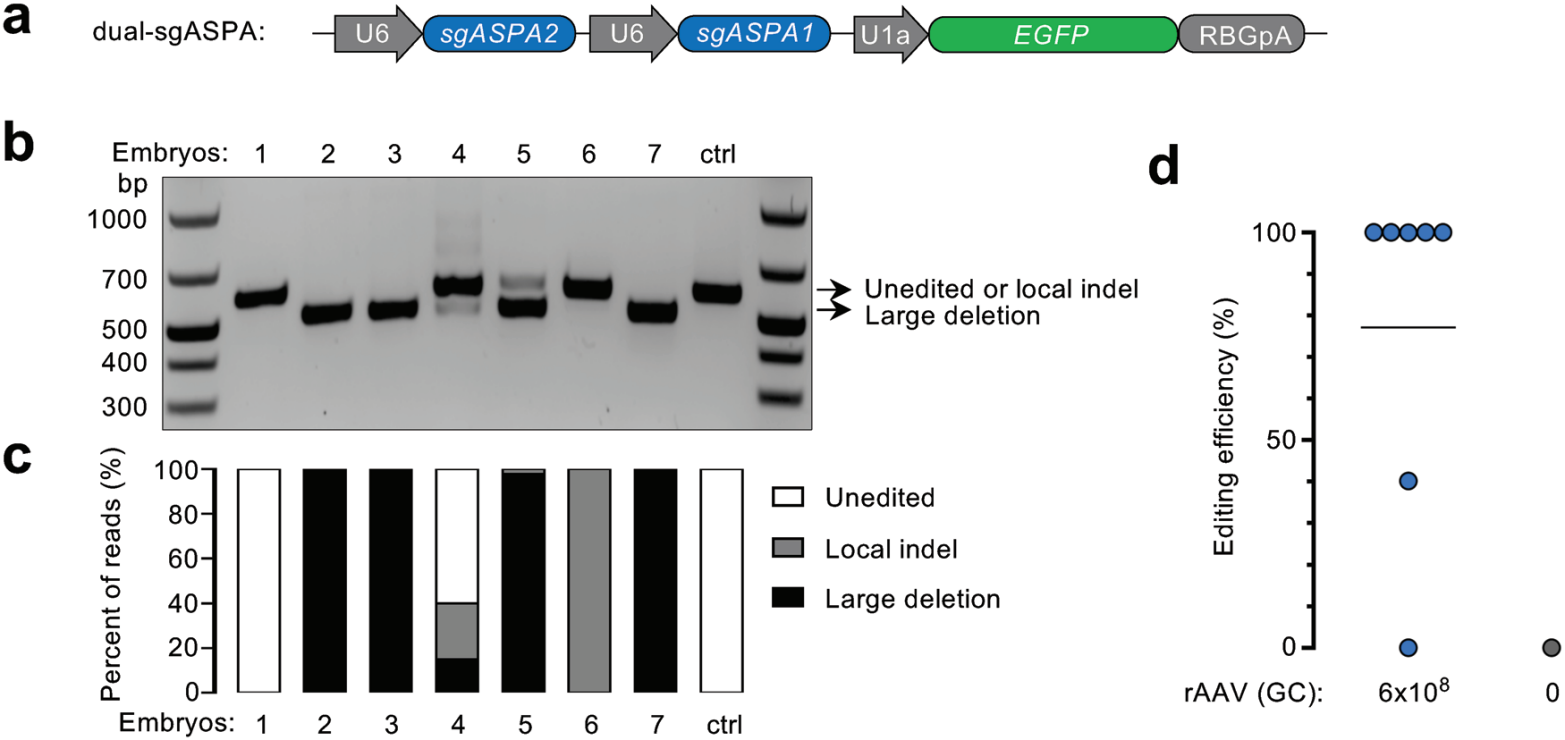
Dual‐guide design achieved efficient genome editing and undetectable off‐targeting. a) Schematic showing the construct expressing two sgRNAs. b) PCR assay revealing large deletions in the *ASPA* gene in some embryos infected with rAAV6.SpCas9 and scAAV6.dual‐sgASPA. DNA from a naïve embryo served as control (ctrl). c) Characterization of *ASPA* gene editing events in individual embryos by SMRT sequencing. Local indel is defined as indel events at either one or both sgRNA targeting sites. Large deletion is defined as deletion between the two sgRNA targeting sites. d) *ASPA* gene editing efficiency of individual embryos as shown in (b) by SMRT sequencing, counting both local indel and large deletion as editing events. The horizontal bar indicates the average value.

In summary, we successfully used rAAV to transduce cynomolgus monkey preimplantation embryos, and achieved gene editing in embryos and fetuses. Precise gene editing is conceivable by combining with a third rAAV carrying a donor template for homology‐directed repair (HDR) as demonstrated in mouse embryos,^2^, ^8^ or by delivering base editors using rAAV.^9^, ^10^ Although we were not able to obtain any live birth positive for gene editing, it may be a gene‐specific phenomenon. Alternatively, the infection could be further optimized to reduce the rAAV dose for toxicity amelioration. For example, a smaller Cas9 variant and sgRNA genes can be packaged within a single AAV vector,^11^ so that infection can be potentially performed at half of the current dose. Recently, another independent group reported data corroborating our findings in mice and expanded the utility of this technique to rats.^8^ Taken together, this rAAV‐mediated approach offers unparalleled ease and efficiency of gene delivery to preimplantation embryos, and is potentially suitable to a broad range of mammalian species. In addition to gene editing and animal modeling, this method can also facilitate gene function studies in early‐stage mammalian embryos.

## Experimental Section

*Animals*: Healthy female cynomolgus monkeys (*Macaca fascicularis*) of 5–10 years old and weighing between 3.62 and 5.90 kg were used in this study. All animals were housed at a facility accredited by the Association for Assessment and Accreditation of Laboratory Animal Care. All animal protocols were approved in advance by the Institutional Animal Care and Use Committee of Yunnan Key Laboratory of Primate Biomedical Research.

*Animal Procedures and Preparation of Zygotes*: Superovulation, collection of oocytes, fertilization, and embryo culture were performed as previously described.^12^, ^13^, ^14^ In brief, healthy female cynomolgus monkeys with regular menstrual cycles were selected as oocyte donors for superovulation. Animals were intramuscularly injected with recombinant human follicle stimulating hormone (rhFSH; Gonal‐f, Merck Serono) for eight consecutive days and with recombinant human chorionic gonadotropin (rhCG; OVIDREL, Merck Serono) on the ninth day. Oocytes were collected by laparoscopic follicular aspiration (STORZ) 32–35 h after rhCG administration and classified as prophase I (PI; intact germinal vesicle), metaphase I (MI; no germinal vesicle, no polar body), or metaphase II (MII; one polar body). Only MII oocytes were used to perform intracytoplasmic sperm injection. Adult male cynomolgus monkeys of 7–10 years old with fertility were chosen as sperm donors. Semen collection was performed by electroejaculation. The fertilization was confirmed by the presence of two pronuclei. Embryos from 1‐cell stage to blastocyst were cultured in chemically defined, protein‐free hamster embryo culture medium‐9 (HECM‐9) containing 10% fetal calf serum under 37 °C with 5% CO_2_ to allow embryo development.

*AAV Vectors*: For the AAV vectors expressing enhanced green fluorescent protein, the self‐complementary (sc) recombinant AAV genome contained a cytomegalovirus enhancer fused with the chicken beta‐actin promoter (CMV/CB) driving the EGFP gene. The same genome was packaged into various AAV serotype capsids including AAV6, AAV7, AAVrh.39, AAVrh.43, and AAV6.2. The single‐stranded (ss) AAV6 vector expressing *Streptococcus pyogenes* Cas9 (rAAV6.SpCas9) carried the U1a promoter driving SpCas9. The scAAV6 vector expressing a single sgRNA (scAAV6.sgASPA) contained the U6 promoter driving sgRNA, and the CMV/CB driving EGFP. The scAAV6 vector expressing two sgRNAs (scAAV6.Dual‐sgASPA) contained two U6‐sgRNA cassettes in tandem, and the U1a promoter driving EGFP. All AAV vectors were produced by triple transfection in HEK293T cells and purified by two rounds of CsCl sedimentation. The titers were determined by droplet digital PCR (ddPCR, Bio‐Rad), and the purity was assessed by protein gel electrophoresis and silver staining.

*Infection of Zygotes with AAV Vectors*: Zygotes obtained by ICSI were maintained in HECM‐9 medium for 4 h until infection. No more than 10 zygotes were placed in a droplet of 60 µL of HECM‐9 medium containing various amounts of AAV vectors as indicated. Zygotes were incubated with AAV vectors for various durations as indicated, rinsed, and cultured in HECM‐9 medium for either seven days for microscopy and DNA analysis, or for one day followed by transfer to recipients.

*Cell Culture and Transfection*: COS‐7 cells (ATCC, Cat. No. CRL‐1651) were maintained in Dulbecco's Modified Eagle Media (DMEM, Thermo Fisher Scientific, Cat. No. 11965084) with 10% fetal bovine serum (FBS, Thermo Fisher Scientific, Cat. No. 10438034) under standard cell culture condition. COS‐7 cells were split into 6‐well plates to obtain ≈80% density for transfection the next day. Transfection was performed using Lipofectamine 2000 (Thermo Fisher Scientific, Cat. No. 11668‐019) with 0.8 µg of the plasmid expressing SpCas9 and 0.8 µg of a plasmid expressing one or two sgRNAs (1.6 µg of total plasmid DNA per well).

*DNA Preparation*: For COS‐7 cells, genomic DNA was isolated three days after transfection using the QIAmp DNA Mini Kit (Qiagen, Cat. No. 51306). The same kit was used to extract genomic DNA from animal tissues. For embryos cultured for seven days ex vivo, individual embryos were subjected to whole‐genome amplification using the REPLI‐g Single Cell Kit (Qiagen, Cat. No. 150343).

*T7EI Nuclease Assay*: A portion of the genomic sequence harboring a sgRNA targeting site was amplified using the KOD Hot Start DNA Polymerase (EMD Millipore, Cat. No. 71086), and purified using the QIAquick PCR purification kit (Qiagen, Cat. No. 28106). Approximately 350 µg of purified PCR product was denatured and reannealed as described previously,^15^ and incubated with 10 units of T7EI nuclease (NEB, Cat. No. M0302L) under 37 °C for 30 min. The reaction was quenched by adding 0.75 µL of 0.5 m EDTA. The entire reaction mixture was resolved on a 2% agarose gel containing ethidium bromide and imaged.

*TOPO Sequencing*: Purified PCR product was cloned into the pCR‐Blunt II‐TOPO vector using the Zero Blunt TOPO PCR Cloning Kit (Thermo Fisher Scientific, Cat. No. K280002), and transformed into DH5*α Escherichia coli* bacteria. Plasmid of individual colonies was isolated using the QIAprep Spin Miniprep Kit (Qiagen, Cat. No. 27106), and sequenced by Sanger method.

*Detection of rAAV Genome Integration by PCR*: The pAAV.SpCas9 plasmid was linearized by SacII digestion, purified using the QIAquick PCR Purification Kit (Qiagen, Cat. No. 28106), and quantified by Qubit 3.0 Fluorometer (Thermo Fisher Scientific). Linearized plasmid was 10× serially diluted and used as template in PCR to determine assay sensitivity. Each PCR also contained 100 ng of human genomic DNA as background. 100 ng of monkey tissue DNA was subjected to the same PCR to detect rAAV genome. To specifically assess ITR integration, a portion of Cynomolgus *ASPA* genomic DNA containing the sgASPA target site was modified to contain a SbfI site between the 3rd and 4th bps upstream of the PAM, synthesized (gBlock, IDT), and cloned into pcDNA3.1 between the MluI and XhoI sites to yield pcDNA.CynoASPA‐SbfI. The pAAV.SpCas9 was digested with SbfI, and the fragment containing the rAAV genome (containing both ITRs) was cloned into pcDNA.CynoASPA‐SbfI to yield pcDNA.CynoASPA.ITR‐U1a‐SpCas9‐ITR. This plasmid was digested using BglII, and the two fragments were used as templates in upstream and downstream ITR integration PCR, respectively. All PCR primers are listed in Figure S9c in the Supporting Information.

*Single‐Molecule Real‐Time Sequencing and Bioinformatics Analysis*: Target amplicons from genomic material for single‐molecule real‐time sequencing was generated by PCR using asymmetrically barcoded primers (Figure S9b, Supporting Information). PCR products were pooled at equimolar ratios for SMRTbell template preparation and sequenced using a PacBio RS II sequencer following standard guidelines and procedures by the University of Massachusetts Medical School, Deep Sequencing Core. Raw reads were processed by SMRT Analysis software (v2.3.0) pipelines to produce reads‐of‐inserts (ROIs) representing multiplexed PCR amplicon sequences in fastq format. All downstream workflows were performed using the Galaxy web‐based platform for genome data analysis,^16^, ^17^, ^18^ unless specified. ROIs were demultiplexed by both 5′ and 3′ barcodes so that only full and intact reads that encompassed both asymmetric barcodes were considered for analysis. Filtered sequences were then aligned with BWA‐MEM^19^ to custom references. For embryos treated with a single sgRNA, the reference used was the unedited, wild‐type target amplicon sequence of the cynomolgus monkey *ASPA* gene. Mapped reads exhibiting imperfect alignment (deletions, insertions, and mismatches) across the predicted edit site (−3 nt of the PAM) were designated as editing events. For embryos treated with two sgRNAs, which may result in a 76 bp deletion, reads were aligned to a fasta file containing a full‐length amplicon sequence and a sequence that lacked the 76 nucleotides between the two anticipated cut sites. This method improved mapping accuracy. Reads mapping to the full‐length sequence as imperfect alignments (deletions, insertions, and mismatches) across the predicted edit site (−3 nt of the PAM) were designated as editing events resulting in local indel events. Reads mapping to the reference containing the 76 nt deletion, were considered large deletion events. To tabulate the number of editing events in each sample, mapped reads where converted to fasta format and were clustered with USEARCH v8.1 sequence analysis tools.^20^ Specifically, identical sequences were tabulated with the ‐derep_fulllength command, followed by sequence clustering using operational taxonomic units (OTU) with the ‐cluster_otus command with the following options: ‐fulldp, ‐otu_radius_pct 0.1, ‐minsize 5, ‐gapopen *I/1.0E, and ‐gapext *I/0.5E. Sequence clusters were manually curated to group and count unique indel types. Editing prevalence was scored as a percentage of total reads.

*Bioinformatic Prediction of Candidate Off‐Targeting Sites*: The web‐based tool Cas‐OFFinder (http://www.rgenome.net/cas-offinder/)^21^ was used to predict candidate off‐targeting sites of sgASPA1 and sgASPA2 in the cynomolgus monkey genome. The search parameters were set as: PAM type was “SpCas9 from *Streptococcus pyogenes*: 5′‐NGG‐3′”; target genome was “*Macaca fascicularis* (5.0)—Crab‐eating macaque”; mismatch number was equal or less than 3. Query Sequences were “GATTCAGAGAACAGGGCTGG” for sgASPA1, and “CTATCTTTGGAGGAACCCAT” for sgASPA2. In total, 35 OT sites were predicted for sgASPA1, and 8 OT sites were predicted for sgASPA2.

## Conflict of Interest

G.G. is a scientific co‐founder of Aspa Therapeutics and Voyager Therapeutics and holds equity in the companies. G.G. is an inventor of patents related to adeno‐associated virus (AAV) gene therapy with potential royalties licensed to Aspa Therapeutics, Voyager Therapeutics, and other biopharmaceutical companies.

## Supporting information

Supplementary

## References

[1] Y. Niu , B. Shen , Y. Cui , Y. Chen , J. Wang , L. Wang , Y. Kang , X. Zhao , W. Si , W. Li , A. P. Xiang , J. Zhou , X. Guo , Y. Bi , C. Si , B. Hu , G. Dong , H. Wang , Z. Zhou , T. Li , T. Tan , X. Pu , F. Wang , S. Ji , Q. Zhou , X. Huang , W. Ji , J. Sha , Cell 2014, 156, 836. 24486104 10.1016/j.cell.2014.01.027

[2] Y. Yoon , D. Wang , P. W. L. Tai , J. Riley , G. Gao , J. A. Rivera‐Pérez , Nat. Commun. 2018, 9, 412. 29379011 10.1038/s41467-017-02706-7 PMC5788975

[3] R. Kaul , G. P. Gao , K. Balamurugan , R. Matalon , Nat. Genet. 1993, 5, 118. 8252036 10.1038/ng1093-118

[4] S. S. Ahmed , H. Li , C. Cao , E. M. Sikoglu , A. R. Denninger , Q. Su , S. Eaton , A. A. Liso Navarro , J. Xie , S. Szucs , H. Zhang , C. Moore , D. A. Kirschner , T. N. Seyfried , T. R. Flotte , R. Matalon , G. Gao , Mol. Ther. 2013, 21, 2136. 23817205 10.1038/mt.2013.138 PMC3863789

[5] S. S. Ahmed , S. A. Schattgen , A. E. Frakes , E. M. Sikoglu , Q. Su , J. Li , T. G. Hampton , A. R. Denninger , D. A. Kirschner , B. Kaspar , R. Matalon , G. Gao , Mol. Ther. 2016, 24, 1030. 27039844 10.1038/mt.2016.68 PMC4923332

[6] D. J. Gessler , D. Li , H. Xu , Q. Su , J. Sanmiguel , S. Tuncer , C. Moore , J. King , R. Matalon , G. Gao , JCI Insight 2017, 2, e90807. 28194442 10.1172/jci.insight.90807 PMC5291725

[7] P. D. Hsu , D. A. Scott , J. A. Weinstein , F. A. Ran , S. Konermann , V. Agarwala , Y. Li , E. J. Fine , X. Wu , O. Shalem , T. J. Cradick , L. A. Marraffini , G. Bao , F. Zhang , Nat. Biotechnol. 2013, 31, 827. 23873081 10.1038/nbt.2647 PMC3969858

[8] N. Mizuno , E. Mizutani , H. Sato , M. Kasai , A. Ogawa , F. Suchy , T. Yamaguchi , H. Nakauchi , iScience 2018, 9, 286. 30447647 10.1016/j.isci.2018.10.030 PMC6240711

[9] S.‐M. Ryu , T. Koo , K. Kim , K. Lim , G. Baek , S.‐T. Kim , H. S. Kim , D.‐e. Kim , H. Lee , E. Chung , J.‐S. Kim , Nat. Biotechnol. 2018, 36, 536. 29702637 10.1038/nbt.4148

[10] L. Villiger , H. M. Grisch‐Chan , H. Lindsay , F. Ringnalda , C. B. Pogliano , G. Allegri , R. Fingerhut , J. Häberle , J. Matos , M. D. Robinson , B. Thöny , G. Schwank , Nat. Med. 2018, 24, 1519. 30297904 10.1038/s41591-018-0209-1

[11] F. A. Ran , L. Cong , W. X. Yan , D. A. Scott , J. S. Gootenberg , A. J. Kriz , B. Zetsche , O. Shalem , X. Wu , K. S. Makarova , E. V. Koonin , P. A. Sharp , F. Zhang , Nature 2015, 520, 186. 25830891 10.1038/nature14299 PMC4393360

[12] Y. Chen , Y. Niu , W. Ji , J. Genet. Genomics 2012, 39, 247. 22749011 10.1016/j.jgg.2012.04.007

[13] Y. Chen , Y. Zheng , Y. Kang , W. Yang , Y. Niu , X. Guo , Z. Tu , C. Si , H. Wang , R. Xing , X. Pu , S.‐H. Yang , S. Li , W. Ji , X.‐J. Li , Hum. Mol. Genet. 2015, 24, 3764. 25859012 10.1093/hmg/ddv120 PMC5007610

[14] Y. Niu , Y. Yu , A. Bernat , S. Yang , X. He , X. Guo , D. Chen , Y. Chen , S. Ji , W. Si , Y. Lv , T. Tan , Q. Wei , H. Wang , L. Shi , J. Guan , X. Zhu , M. Afanassieff , P. Savatier , K. Zhang , Q. Zhou , W. Ji , Proc. Natl. Acad. Sci. USA 2010, 107, 17663. 20870965 10.1073/pnas.1006563107 PMC2955145

[15] F. A. Ran , P. D. Hsu , J. Wright , V. Agarwala , D. A. Scott , F. Zhang , Nat. Protoc. 2013, 8, 2281. 24157548 10.1038/nprot.2013.143 PMC3969860

[16] D. Blankenberg , G. Von Kuster , N. Coraor , G. Ananda , R. Lazarus , M. Mangan , A. Nekrutenko , J. Taylor , Curr. Protoc. Mol. Biol. 2010, 89, 19.10.1. 10.1002/0471142727.mb1910s89 PMC4264107 20069535

[17] B. Giardine , Genome Res. 2005, 15, 1451. 16169926 10.1101/gr.4086505 PMC1240089

[18] J. Goecks , A. Nekrutenko , J. Taylor , The Galaxy Team , Genome Biol. 2010, 11, R86. 20738864 10.1186/gb-2010-11-8-r86 PMC2945788

[19] H. Li , R. Durbin , Bioinformatics 2009, 25, 1754. 19451168 10.1093/bioinformatics/btp324 PMC2705234

[20] R. C. Edgar , Bioinformatics 2010, 26, 2460. 20709691 10.1093/bioinformatics/btq461

[21] S. Bae , J. Park , J.‐S. Kim , Bioinformatics 2014, 30, 1473. 24463181 10.1093/bioinformatics/btu048 PMC4016707

