# Supplementary material for: Gene Delivery to Nonhuman Primate Preimplantation Embryos Using Recombinant Adeno‐Associated Virus
Source: Adv Sci (Weinh). 2019 Sep 4;6(21):1900440. doi: 10.1002/advs.201900440 (PMC6839749; doi:10.1002/advs.201900440)
Supplement: Supplementary file 1 — Supplementary [file ADVS-6-1900440-s001.pdf]

Copyright WILEY-VCH Verlag GmbH & Co. KGaA, 69469 Weinheim, Germany, 2019.

Supporting Information

for *Adv. Sci.,* DOI: 10.1002/advs.201900440

Gene Delivery to Nonhuman Primate Preimplantation Embryos Using Recombinant Adeno-Associated Virus

Dan Wang, Yuyu Niu, Lingzhi Ren, Yu Kang, Phillip W. L. Tai, Chenyang Si, Craig A. Mendonca, Hong Ma, Guangping Gao, and Weizhi Ji**

Supplementary Information

Figure S1. Optimizing AAV serotype for NHP embryo transduction.

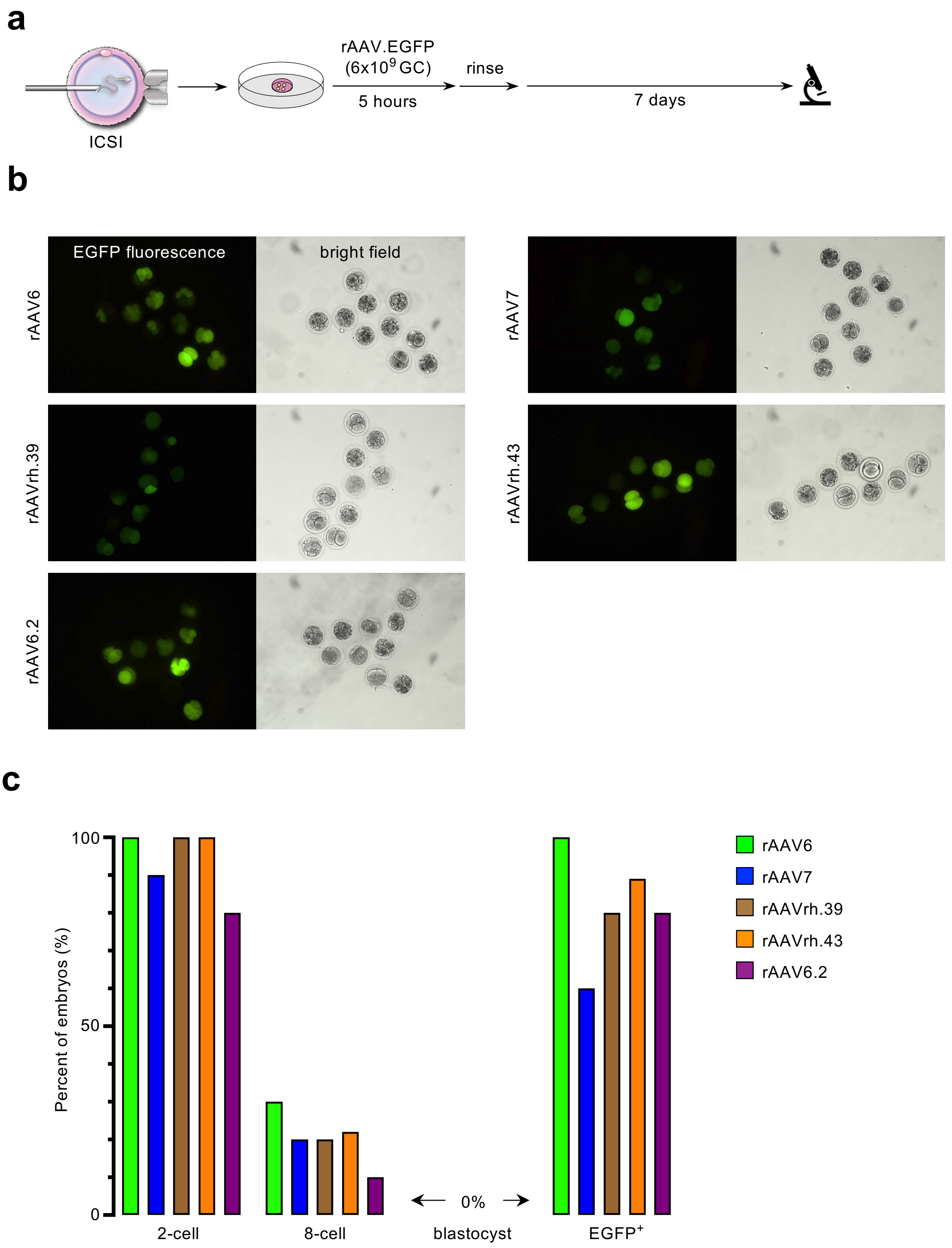

Figure S2. Optimizing infection time with rAAV6.EGFP.

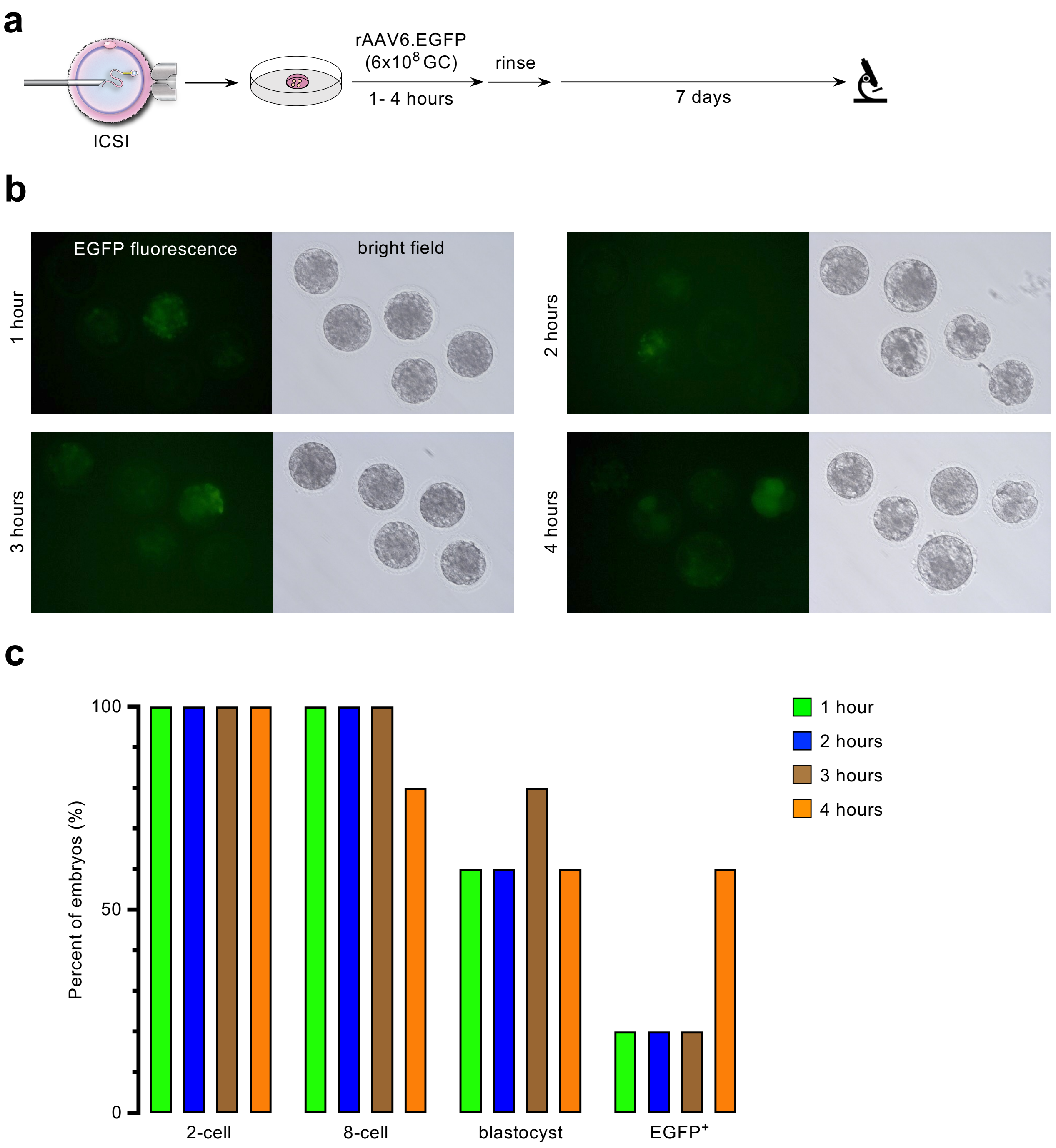

Figure S3. Screening sgRNAs targeting *ASPA* in COS-7 cells by plasmid transfection.

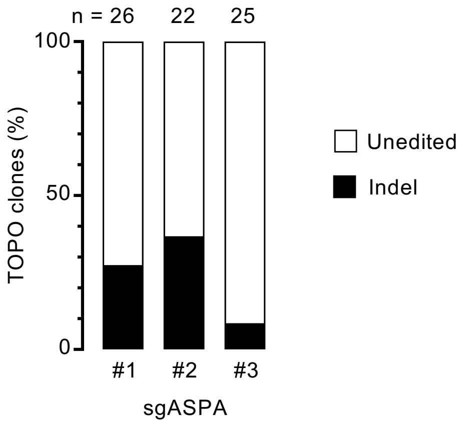

Figure S4. Embryo development and transduction of NHP zygotes after co-infection with rAAV6.SpCas9 and scAAV6.sgASPA2.

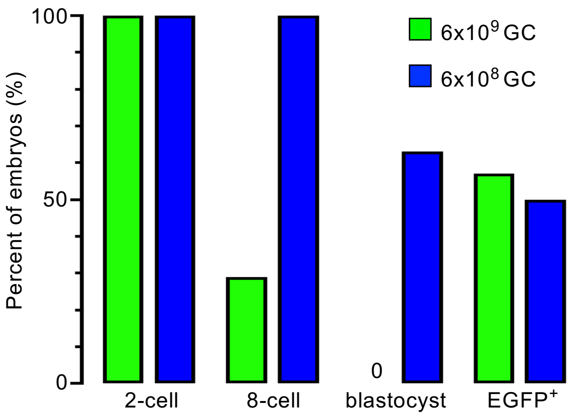

Figure S5. rAAV genome integration was not detected in three aborted fetuses carrying *ASPA* gene editing.

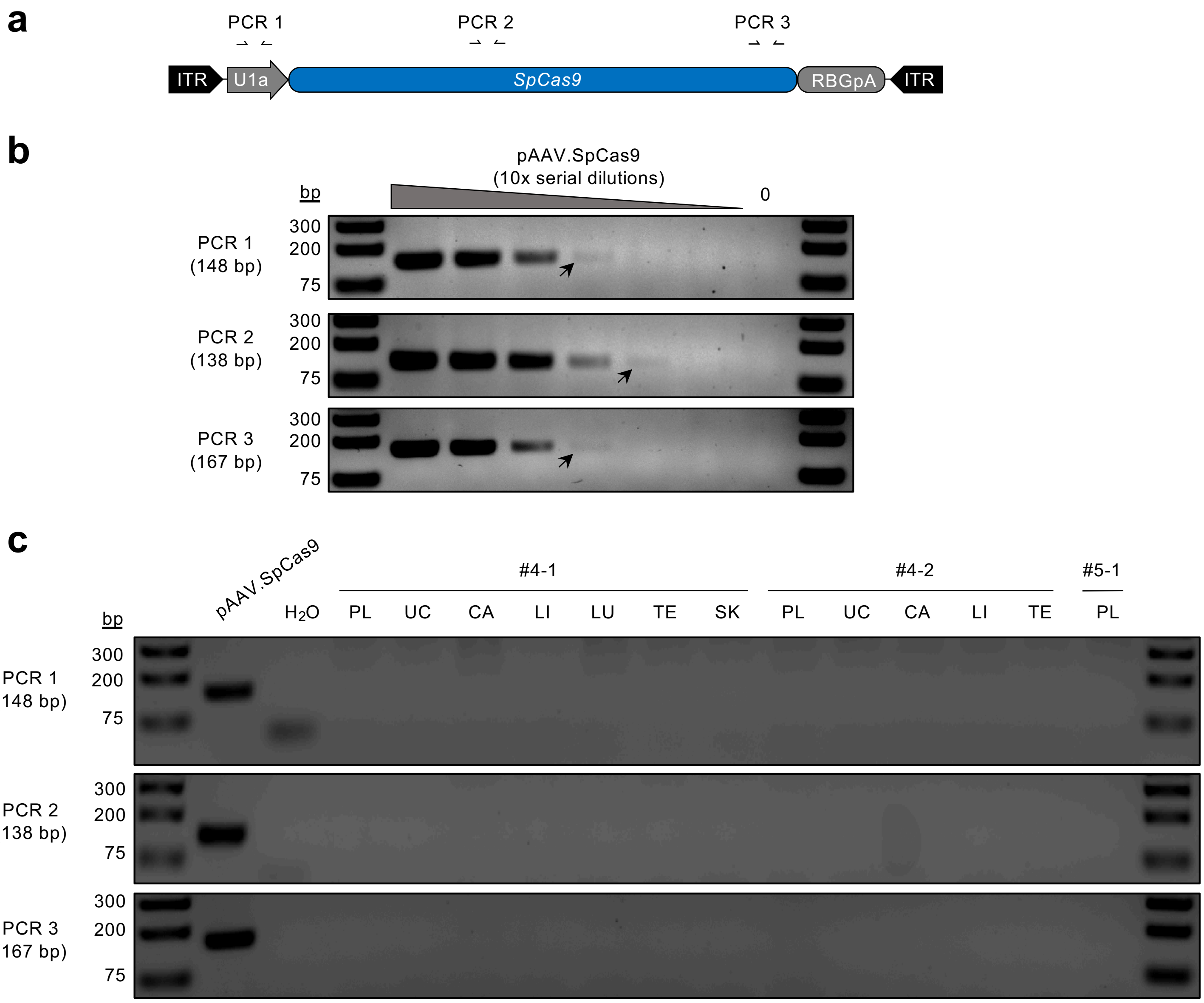

Figure S6. rAAV ITR integration was not detected in three aborted fetuses carrying *ASPA* gene editing.

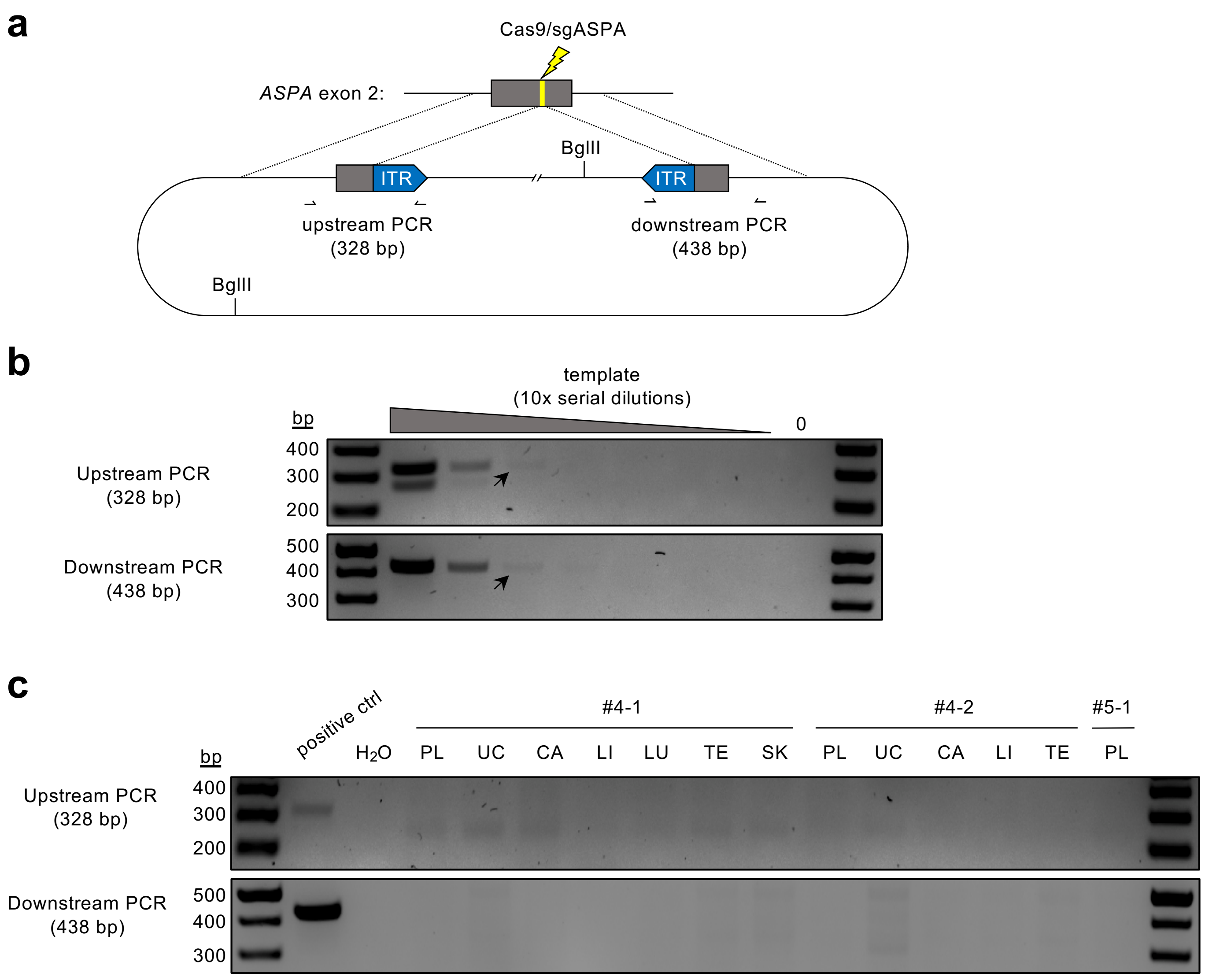

Figure S7. Dual-sgASPA generated local indels and large deletions in COS-7 cells by plasmid transfection.

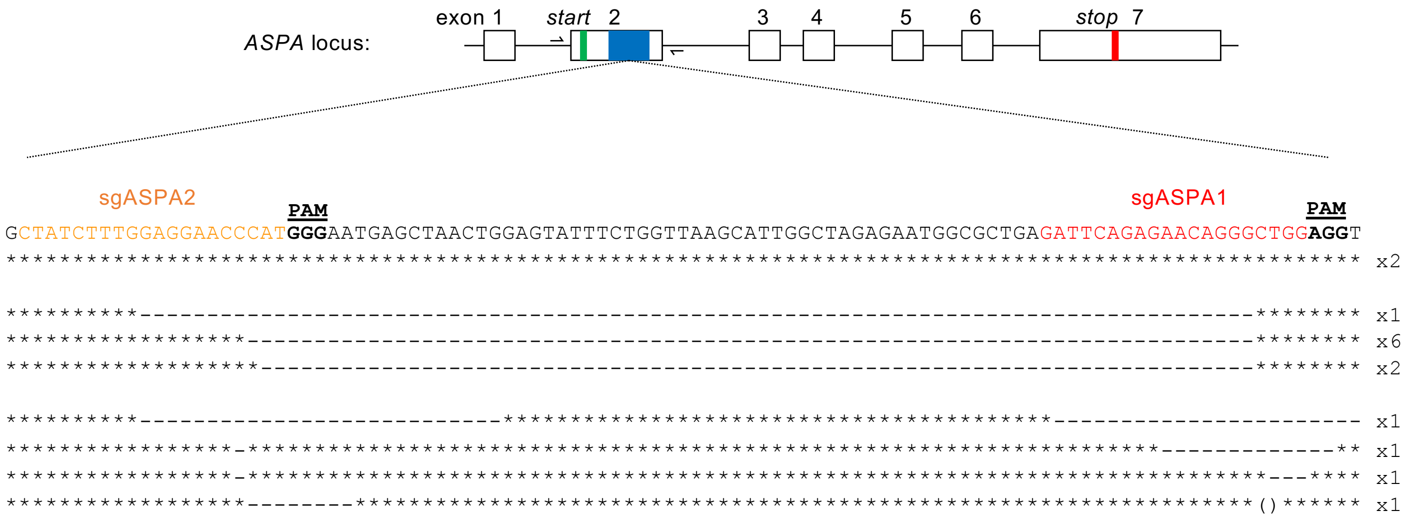

Figure S8. No off-targeting was detected

a

```
                  20     13             1
                  .      .              .  PAM
sgASPA1           GATTCAGA GAACAGGGCTGG AGG
OT1               GATggAGg GAACAGGGCTGG GGG
OT2               GAgagAGA GAACAGGGCTGG AGG
OT3               GtTcCAGt GAACAGGGCTGG GGG

                  20     13             1
                  .      .              .  PAM
sgASPA2           CTATCTTT GGAGGAACCCAT GGG
OT4               CTATCTTT GcAGGgACCCAT GGG
OT5               gTATCaTg GGAGGAACCCAT CGG
OT6               gTgTCTTa GGAGGAACCCAT TGG
```

b

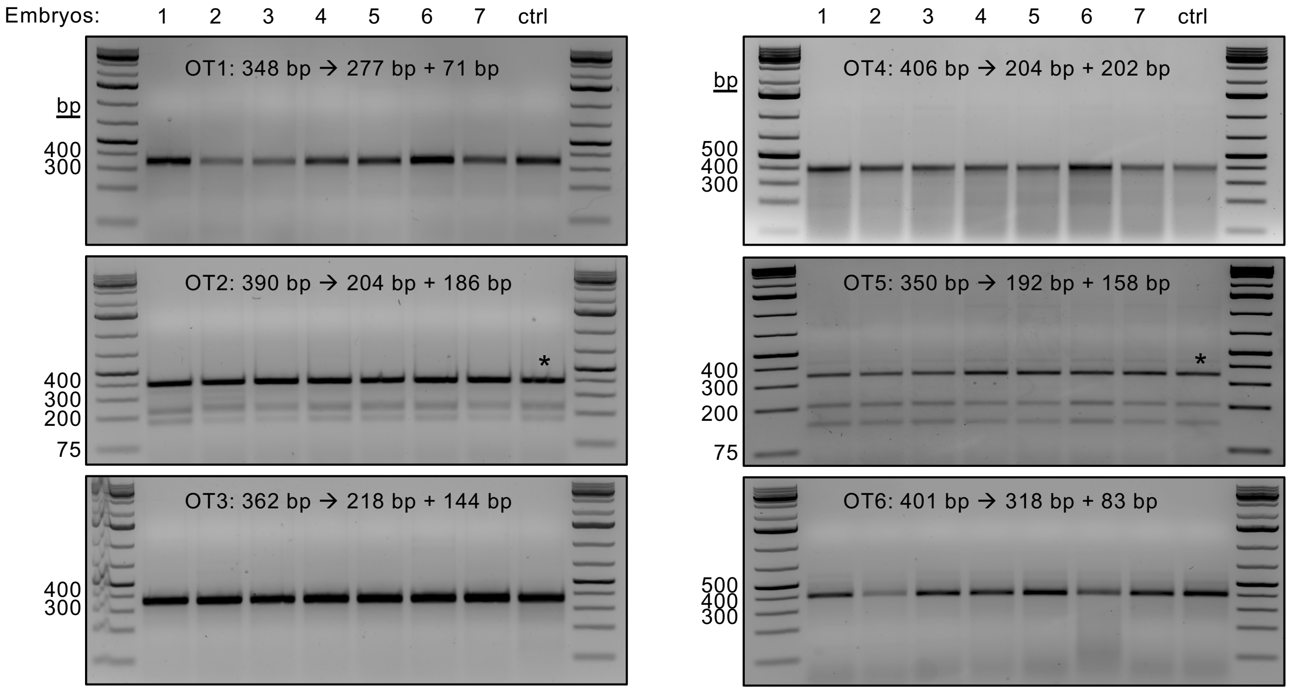

c

```
OT2: ...TAGCT[C/T]CTATA...    390 bp → 218 bp + 172 bp
OT5: ...TGATG[GT/AA]AAATA...  350 bp → 206 bp + 144 bp
```

d

Embryo	OT1 reads		OT2 reads		OT3 reads		OT4 reads		OT5 reads		OT6 reads	
	Total	MM	Total	MM	Total	MM	Total	MM	Total	MM	Total	MM
# 2	2,834	30	5,496	0	4,135	5	n/d	n/d	8,685	0	2,025	0
# 3	6,439	39	5,351	0	6,288	19	n/d	n/d	11,200	0	6,180	0
# 7	10,315	71	6,684	0	7,268	0	n/d	n/d	8,726	0	3,237	0
ctrl	10,037	44	3,873	0	5,110	0	n/d	n/d	10,351	0	3,503	0

e

```
                     PAM
OT1   Ref   ACAGGGC TGGGGGATTCTCTCCC
      MM    ACAGGGC T-GGGGATTCTCTCCC

OT3   Ref   ACAGGGC TGGGGGTGATAACCAG
      MM    ACAGGGC TGGGGGTGATAACCAG
                     ^
                     G
```

Figure S9. Nucleic acid sequences

a

	guide RNA (5' → 3')	PAM
sgASPA1	GAUUCAGAGAACAGGGCUGG	AGG
sgASPA2	CUAUCUUUGGAGGAACCCAU	GGG
sgASPA3	UCCAGUUAGCUCAUUCCCAU	GGG

b

Barcode attached to forward PCR primers (5' → 3')	Barcode attached to reverse PCR primers (5' → 3')
GCGCTCTGTGTGCAGC	AGAGTACTACATATGA
TCATGAGTCGACACTA	CGTGTGCATAGATCGC
TATCTATCGTATACGC	ATGTATCTCGACTGCA
ATCACACTGCATCTGA	GACTCGACGCAGAGTC
ACGTACGCTCGTCATA	CGATGACGTCGCTGTA
TGTGAGTCAGTACGCG	CACACGTAGTCTGCGC
AGAGACACGATACTCA	GCTGTATCGCAGAGAC
CTGCTAGAGTCTACAG	CGAGCTATCTCATACT
AGCACTCGCGTCAGTG	CATGAGTACTCGTCGC
TCATGCACGTCTCGCT	CAGCGACTGTGATACT
AGAGCATCTCTGTACT	TGTCGCATCATATGAT
CGCATCGACTACGCTA	GCTGTGATCTACGTCT
CGTAGCGTGCTATCAC	TGAGTAGCATGACACG
ATGCTGATGACTGCGA	GACATGCAGTCTCACA
TGCGTGAGCTGTACAT	CAGTAGCGCACTGAGC
CGATCATCTATAGACA	CTGCGTGCGCGATAGT

c

Oligo ID	Sequence (5' → 3')	Purpose
DW789	TCTGTACTTTGCCCTTTGGG	T7EI PCR for sgASPA, forward
DW790	CTCTTTCACACACCTACCAC	T7EI PCR for sgASPA, reverse
DW910	AGACAGTGTCCATAAACGGG	PCR to detect large deletions by dual-sgASPA, forward
DW911	CATAGCGACTGCACGTATGG	PCR to detect large deletions by dual-sgASPA, reverse; PCR to detect downstream ITR integration
DW1123	TCTGTACTTTGCCCTTTGGG	PCR to detect upstream ITR integration
DW1033	ATGGTGGGTTAGGAAGGATC	T7EI PCR for OT1, forward
DW1034	GGAATCTGGAGGTTTCTAGC	T7EI PCR for OT1, reverse
DW1035	AGGGAAATCAGAGTGATGCC	T7EI PCR for OT2, forward
DW1036	TGAGTAGGACACATTCTGCC	T7EI PCR for OT2, reverse
DW1037	TTGCCAAGCCAATGAGTTGC	T7EI PCR for OT3, forward
DW1038	GCTGTGTGAACGTCATTTGG	T7EI PCR for OT3, reverse
DW1039	ATTTGCCTCTGCTGTGAGAC	T7EI PCR for OT4, forward
DW1040	GATGAACTTACCCTCCACTG	T7EI PCR for OT4, reverse
DW1041	CCCTATTCCTTACCTTCAGG	T7EI PCR for OT5, forward
DW1042	GACTCACAGTTTCACATGGC	T7EI PCR for OT5, reverse
DW1043	GGTGAAATGGTTTGGCTGTG	T7EI PCR for OT6, forward
DW1044	ATCCATCCATCTGTCCTCTG	T7EI PCR for OT6, reverse
DW1105	GGCGGTACTATGTAGATGAG	PCR to detect U1a promoter, forward
DW1106	TCATGTGTACTGGCTCCCAC	PCR to detect U1a promoter, reverse
DW560	CTGAGCAAGGACACCTACGA	PCR to detect SpCas9 N-half, forward
DW561	CTCGGTGTTCACTCTCAGGA	PCR to detect SpCas9 N-half, reverse
DW1107	ATCTGGACAAAGTGCTGTCC	PCR to detect SpCas9 C-half, forward
DW1108	CTCTTTGGTGCTGGTGTACC	PCR to detect SpCas9 C-half, reverse
AAV2.ITR	AGGAACCCCTAGTGATGGAGT	PCR to detect ITR integration

Table S1. Recipients carrying rAAV-infected embryos and the resulting subjects.

Recipient	Ultrasound at 3 weeks	Ultrasound at 3 months	Note
1	singleton	singleton	1 male was born (#1-1)
2	twins	singleton	1 male was born (#2-1)
3	twins	singleton	mother died at 4 months; 1 fetus was found dead (#3-1)
4	twins	twins	abortion at 2 months (#4-1, #4-2)
5	twins	no fetus was detected	abortion at 1 month (#5-1)

Table S2. Predicted candidate off-target sites for sgASPA1.

ID	DNA	Chromosome	Position	Direction	Mismatches
	GAcTCAaA GAAgAGGGCTGG GGG	chr4	19770339	+	3
	aATaCAGA aAACAGGGCTGG CGG	chr4	64173707	–	3
	GAaTCtGA GAACAGGGCaGG AGG	chr4	80678212	–	3
	GtTgCAGA GgACAGGGCTGG TGG	chr4	150464706	+	3
OT1	GATggAGg GAACAGGGCTGG GGG	chr6	836274	+	3
	GATTCAGg GAggAGGGCTGG CGG	chr6	66866557	–	3
	GATTCAGA GAgCAGtGCTGt GGG	chr6	108988114	–	3
OT2	GAgagAGA GAACAGGGCTGG AGG	chr6	131017018	+	3
OT3	GtTcCAGt GAACAGGGCTGG GGG	chr8	65766286	+	3
	GATTCAGA GAgCcGGGgTGG GGG	chr7	28171103	–	3
	GATTCtGA GAgCAGGtCTGG TGG	chr7	52468057	–	3
	GATTCAtA GAAaAaGGCTGG AGG	chr7	72950993	+	3
	GccTCAGA GAACAGGGCaGG TGG	chr7	157624232	+	3
	GATTCtGA GAcCAaGGCTGG AGG	chr13	81479736	+	3
	GAgTCAGA GAtCAtGGCTGG GGG	chr13	84026032	+	3
	GgTTCAGA GAACAaaGCTGG GGG	chrX	123043120	–	3
	GAaTtAGA GAACAGGcCTGG TGG	chr11	30598086	+	3
	GAgTgAGA GAAgAGGGCTGG GGG	chr11	87198091	+	3
	GAcTCAGA GAAgAGGaCTGG GGG	chr14	3367753	+	3
	GcTTCAGA GAACtGGGgTGG GGG	chr14	107892812	–	3
	GgTgCAGA GAACAGGGtTGG GGG	chr10	38660891	+	3
	GgTgCAGA GAACAGGGtTGG GGG	chr10	38533862	–	3
	GcTTCAGA GAACAGGGCctG GGG	chr15	2622339	–	3
	GtTTCAGA GcACAGGGgTGG AGG	chr15	38772274	+	3
	GcTTCAGA GAACAGtcCTGG AGG	chr1	42347196	+	3
	aATTCAGA GAAgAGaGCTGG AGG	chr1	71982464	–	3
	aATcCAGA GAcCAGGGCTGG AGG	chr1	180077835	+	3
	GATTCAaA GAACAGGGtTtG GGG	chr1	213447812	+	3
	GAgTCcGA GAACgGGGCTGG CGG	chr16	3839756	+	3
	GATTCAGA GAAaAGtGCaGG TGG	chr16	12436318	+	3
	GgTaCAGA GAAgAGGGCTGG GGG	chr3	27822888	–	3
	GtTaCAGA GAACAGGGCTtG GGG	chr3	162194476	+	3
	GATTCAGg GAgCAGGGCTGG GGG	chr3	163668907	–	2
	GATTCAGA GAACAtGagTGG TGG	chr12	13472873	–	3
	GATTCAGA GAACAGGctTGc TGG	chr12	103024712	–	3

Table S3. Predicted candidate off-target sites for sgASPA2.

ID	DNA	Chromosome	Position	Direction	Mismatches
	CTtTCTTT GGAGGAACCCca GGG	chr2	2780188	+	3
	CTgTCTTg GGAGGAACCCAg TGG	chr2	190402030	–	3
	CTATtTTT GGAGGgACCgAT AGG	chrX	22147135	+	3
	CTATtTgT GGAGGAAgCCAT TGG	chr18	6148992	+	3
OT4	CTATCTTT GcAGGgACCCAT GGG	chr11	128420847	+	2
OT5	gTATCaTg GGAGGAACCCAT CGG	chr5	154556234	+	3
	CTAaCTTT GGAGaAgCCCAT GGG	chr10	93801891	–	3
OT6	gTgTCTTa GGAGGAACCCAT TGG	chr12	110692388	+	3
